# Isolation of a High Affinity Neutralizing Monoclonal Antibody against 2009 Pandemic H1N1 Virus That Binds at the ‘Sa’ Antigenic Site

**DOI:** 10.1371/journal.pone.0055516

**Published:** 2013-01-31

**Authors:** Nachiket Shembekar, Vamsee V. Aditya Mallajosyula, Arpita Mishra, Leena Yeolekar, Rajeev Dhere, Subhash Kapre, Raghavan Varadarajan, Satish Kumar Gupta

**Affiliations:** 1 Reproductive Cell Biology Laboratory, National Institute of Immunology, Aruna Asaf Ali Marg, New Delhi, India; 2 Molecular Biophysics Unit, Indian Institute of Science, Bangalore, India; 3 Serum Institute of India Limited, Pune, India; Centers for Disease Control and Prevention, United States of America

## Abstract

Influenza virus evades host immunity through antigenic drift and shift, and continues to circulate in the human population causing periodic outbreaks including the recent 2009 pandemic. A large segment of the population was potentially susceptible to this novel strain of virus. Historically, monoclonal antibodies (MAbs) have been fundamental tools for diagnosis and epitope mapping of influenza viruses and their importance as an alternate treatment option is also being realized. The current study describes isolation of a high affinity (*K*_D_ = 2.1±0.4 pM) murine MAb, MA2077 that binds specifically to the hemagglutinin (HA) surface glycoprotein of the pandemic virus. The antibody neutralized the 2009 pandemic H1N1 virus in an *in vitro* microneutralization assay (IC_50_ = 0.08 µg/ml). MA2077 also showed hemagglutination inhibition activity (HI titre of 0.50 µg/ml) against the pandemic virus. In a competition ELISA, MA2077 competed with the binding site of the human MAb, 2D1 (isolated from a survivor of the 1918 Spanish flu pandemic) on pandemic H1N1 HA. Epitope mapping studies using yeast cell-surface display of a stable HA1 fragment, wherein ‘Sa’ and ‘Sb’ sites were independently mutated, localized the binding site of MA2077 within the ‘Sa’ antigenic site. These studies will facilitate our understanding of antigen antibody interaction in the context of neutralization of the pandemic influenza virus.

## Introduction

The 2009 H1N1 swine origin influenza A virus (S-OIV, hereafter referred to as pandemic H1N1) is a novel reassortant strain of influenza virus which has gene segments originating from swine, avian and human influenza A viruses and is immunologically distinct from the seasonal H1N1 strains circulating before 2009 [1]. Till date, pandemic H1N1 has caused more than 1.4 million infections with about 25,000 deaths worldwide [2]. Annually updated seasonal tri-valent vaccine is the major prophylactic treatment option for influenza virus infection. However, seasonal influenza vaccines used before 2009, produce low or no cross-reactive antibodies against pandemic H1N1. Moreover, it has been observed that persons below the age of 30 had low cross-reactive antibody titers against pandemic H1N1 [3]. Antiviral drugs such as, oseltamivir are the sole therapeutic option for treating pandemic H1N1 infection. In addition, rapid diagnosis establishing the causative agent and identification of strain is crucial in a pandemic scenario. Owing to the novelty of this 2009 H1N1 S-OIV and susceptibility of large population, it is imperative to seek alternate diagnostic and treatment modalities.

Neutralizing antibodies against the major surface glycoprotein of the influenza virus, hemagglutinin (HA), is the primary correlate of protection in humans [4]. Four major antigenic sites have been mapped onto the H1 HA and antibodies to each of which can neutralize the infectivity of the virus. Two immunodominant sites (Sa and Sb) are located proximal to the receptor binding pocket and elicit high potency neutralizing antibodies. The other two antigenic sites (Ca and Cb) are located at the subunit interface and esterase domain, respectively [5]–[7]. The H1N1 virus caused a deadly pandemic in 1918 (Spanish flu), killing 20–40 million people worldwide [8]. Though the descendants of this 1918 influenza H1 strains have been circulating in humans, they have antigenically diverged from the 1918 virus, including at the four major antigenic sites. Interestingly, 1918 and 2009 H1N1 viruses share a nearly identical ‘Sa’ antigenic site on HA [9], [10].

Specific high affinity antibodies that will enable sensitive detection of the 2009 pandemic H1N1 virus, differentiating it from other seasonal H1N1 strains, are useful. Moreover, antibodies neutralizing pandemic H1N1 can be useful for passive therapeutic purposes. In this direction, we report here the isolation of a novel, high affinity neutralizing mouse monoclonal antibody (MAb) against the 2009 pandemic H1N1-virus. The epitope of this MAb has been mapped onto the ‘Sa’ antigenic site.

## Materials and Methods

### Cell lines, antigens and virus

Madine Darby Canine Kidney (MDCK) cell line (CCL-34) was obtained from American Type Culture Collection, Manassas, VA, USA and cultured in Dulbecco's modified Eagle's medium (DMEM) (Sigma Aldrich Inc., St. Louis, MO, USA). Sp2/O mouse myeloma cell line was obtained from National Centre for Cell Science, Pune, India and cultured in RPMI-1640 medium (Sigma Aldrich Inc.). Media were supplemented with 10% fetal bovine serum (FBS) and an antibiotic-antimycotic cocktail [Penicillin (100 units/ml), Streptomycin (100 µg/ml) and Amphotericin B (0.25 µg/ml); Biological Industries, Kibbutz beit, Haemek, Israel]. Both cell lines were cultured at 37°C under humidified conditions with 5% CO_2_.

Pandemic H1N1 NYMCX-179A (A/California/07/2009: Influenza virus infectious NYMCX-179A, NIBSC Code 09/124) and seasonal H1N1 (A/Solomon Islands/03/2006: Influenza virus infectious IVR-145, NIBSC code 07/144) were received from NIBSC, UK and passaged in MDCK cells in presence of TPCK-trypsin (2 µg/ml; Sigma Aldrich Inc.). Titer of the virus stock was calculated using Reed and Muench method [11].

### Generation of monoclonal antibodies (MAbs)

Female BALB/cJ mice (8–10 weeks old, Small Animal Experimental Facility, National Institute of Immunology, New Delhi, India) were immunized subcutaneously with 7.5 µg equivalent of HA of inactivated, alum adsorbed strain of H1N1 (A/California/07/2009) (Serum Institute of India Limited, Pune, India). Animals were boosted intraperitoneally two times at 4 week intervals with the same amount of the antigen. The animals were kept in the conventional containment conditions and fed *ad libitum*. Immunized animals were used to generate MAbs as per the protocol described elsewhere [12]. In brief, 4–6 weeks after the second booster, the mouse showing highest antibody titres against inactivated pandemic H1N1 antigen in ELISA, was administered intraperitoneally 2 boosters, 7.5 µg equivalent of HA of pandemic H1N1, on consecutive days. On the 4^th^ day, the mouse was euthanized by administration of ketamine-HCl (75 mg/kg) and spleen was harvested aseptically. Splenocytes (12×10^6^) thus obtained were fused with Sp2/O myeloma cells in 2∶1 ratio using 50% polyethylene glycol (Sigma Aldrich Inc.) and plated in eight 24-well cell culture plates (Greiner Bio-one, GmbH, Frickenhausen, Germany) seeded with peritoneal macrophages (10^5^/well) on the previous day. The hybrid cells were selected by growing the fused cells in HAT selection medium (Sigma Aldrich Inc.). Hybrid cell clones secreting MAbs reacting with pandemic H1N1 were identified by screening culture supernatants in ELISA as described later in this section and single cell clones were obtained by 2–3 rounds of limiting dilution. Specific hybrid cell clone secreting MAb neutralizing pandemic H1N1 virus was grown as ascites in BALB/cJ mice primed with Pristane (2, 6, 10, 14 – tetramethylpentadecane, Sigma Aldrich Inc.). The antibody was purified from ascites using Protein-G Sepharose (GE Healthcare Biosciences AB, Uppsala, Sweden) as per the manufacturer's instructions. Isotyping of the antibodies was carried out using murine MAb isotyping kit (Sigma Aldrich Inc.) by indirect ELISA as per the instructions provided with the kit.

### Ethics statement

All the experiments were performed after due approval from Institutional Animal Ethical Committee, National Institute of Immunology, New Delhi (IAEC#247/10) and following its guidelines.

### ELISA reactivity studies

Briefly, 96-well plates (Greiner Bio-one) were coated with beta-propiolactone (BPL)- inactivated pandemic H1N1 virus (A/California/07/2009) (Serum Institute of India Limited) (200 ng/well) and Vaxigrip [2009–10 season, Sanofi Pasteur SA, Val de reuil, France (comprising of H1N1 A/Brisbane/59/2007, H3N2 A/Brisbane/10/2007 and B/Brisbane/60/2008)] (equivalent to 200 ng total HA protein per well). Microtiter plates coated with ovalbumin (200 ng/well) were used as negative control. In addition, mammalian-expressed recombinant HA (rHA) protein of pandemic H1N1 (A/California/04/2009) and rHA of seasonal H1N1 (A/Solomon Islands/03/2006) (160 ng/well; Sino Biological Inc., Beijing, China) were also immobilized. Coating was performed at 37°C for 1 h followed by overnight incubation at 4°C. Following day, plates were blocked with 1% BSA or 1% milk protein in phosphate buffer saline (PBS) (pH 7.4) for 2 h at 37°C. After washing the plates with PBS containing 0.05% Tween-20 (PBST), 100 µl of cell culture supernatants or purified MAb MA2077 at varying concentration (10 µg/ml to 0.1 ng/ml) were added and plates were incubated for 1 h at 37°C. After 3 rounds of washing with PBST, HRP conjugated goat-anti-mouse antibody (Pierce Biotechnology Inc., Rockford, IL, USA) was added at 1∶10000 dilution and incubated for 1 h at 37°C. Following 3 washes with PBST, the reaction was developed using 0.5 mg/ml o-phenylene-diamine (OPD) (Sigma Aldrich Inc.) as substrate along with 0.06% hydrogen peroxide (Merck, Mumbai, India) in 0.1M citrate-phosphate buffer (pH 5). The reaction was stopped with 50 µl 5N H_2_SO_4_ and optical density was measured at 490 nm with a reference wavelength of 630 nm.

### Binding affinity studies using Surface Plasmon Resonance (SPR)

The binding affinity of MAb MA2077 to rHA of pandemic H1N1 A/California/04/2009 (Sino Biological Inc.) was determined by Surface Plasmon Resonance (SPR) experiments performed with a Biacore 2000 optical biosensor (Biacore, Uppsala, Sweden) at 25°C. Eight hundred resonance units (RU) of MA2077 was immobilized by standard amine coupling to the surface of a research-grade CM5 chip (GE HealthCare, Uppsala, Sweden). A sensor channel immobilized with ovalbumin served as a negative control for each binding interaction. Five different concentrations of the pandemic H1N1 A/California/04/2009 rHA or H1N1 A/PR/8/34 rHA trimer (Sino Biological Inc) in the range between 0.75–5.5 nM were passed over each channel in a running buffer of PBS (pH 7.4) containing 0.05% P20 surfactant. Both binding and dissociation were measured at a flow rate of 30 µl/min. After every binding event, the sensor surface was regenerated by repeated washes with 4M MgCl_2_ at a flow rate of 100 µl/min. Each binding curve was analyzed after correcting for non-specific binding by subtraction of the signals obtained from the negative-control flow channel. The kinetic parameters were obtained by fitting the data to the simple 1∶1 Langmuir interaction model using BIA EVALUATION 3.1 software.

### Microneutralization assay

Microneutralization assay was performed using MDCK cell line as per the WHO guidelines [13]. MDCK cells (15000/well) were seeded in 96-well cell culture plate (Greiner Bio-one) and incubated overnight under standard culture conditions. The next day, an equal volume of antibody and 100 TCID_50_ units of the virus were incubated for 1 h at room temperature. After 1 h, virus-antibody mixture was added onto the seeded MDCK cells and plates were further incubated for 24 h at 32°C under humidified atmosphere and 5% CO_2_. Subsequently, cells were fixed with 80% acetone and probed with 1∶4000 dilution of anti-influenza A–nucleoprotein antibody (Millipore, Billerica, MA, USA). After 3 rounds of washing with PBST, the plates were incubated with 1∶10000 dilution of HRP conjugated goat-anti-mouse antibody (Pierce Biotechnology Inc.) and incubated for 1 h at 37°C. After washing three times with PBST, reaction was developed as mentioned above for ELISA. The fifty percent inhibitory concentration (IC_50_) of the neutralizing antibody was calculated using the non-linear regression program of GraphPad Prism software. The specific neutralizing activity of the MAb was calculated as the lowest concentration of the antibody that showed 10% neutralization of 100 TCID_50_ units of the virus.

### Hemagglutination inhibition (HI) assay

HI assay was performed according to the WHO guidelines using four HA units of the influenza virus and 1% guinea pig RBCs [13]. The specific HI activity (HI titre) of the MAb represents the lowest concentration of the antibody showing HI activity.

### Epitope mapping

#### i) Competition ELISA

Competition ELISA was carried out as described elsewhere [14]. Briefly, 96-well half area plates (Corning Incorporated, NY, USA) were coated with 125 ng of pandemic H1N1 A/California/04/2009 rHA in PBS, overnight at 4°C. Plates were then washed 3 times with PBST and blocked for 1 h with 200 µl of 1% BSA in PBST. After washing, 25 µl of MAb 2D1 was added to each well at 2 µg/ml concentration followed by a 4-fold serial dilution. After 2 h of incubation with 2D1, the solution in each well was replaced by 25 µl of MA2077 at a fixed concentration (1 ng/ml) as determined from the titration curve of MA2077 with pandemic H1N1 rHA. The experiment was carried out in quadruplicates. After 2 h of incubation with MA2077, the plates were washed with PBST. The wells were then probed with appropriate secondary antibodies. Two sets of wells were probed with 25 µl of alkaline phosphatase (ALP)-conjugated goat anti-mouse antibody (Sigma Aldrich Inc.) at a predetermined dilution (1∶10000) and incubated at room temperature for 2 h. Alternatively, the other two sets of wells were probed with 25 µl of ALP-conjugated goat anti-human antibody (Sigma Aldrich Inc.) at a predetermined dilution (1∶10000). The plates were washed and developed using the chromogenic substrate p-nitrophenyl phosphate (Sigma Aldrich Inc.). The optical density was measured at 405 nm (SPECTRAmax Plus 384, Molecular Devices, USA). MAb 2D1 binding to rHA of pandemic H1N1 in a direct-binding ELISA (at the same concentrations used for the competition ELISA) was also carried out.

#### ii) Construction of yeast surface display vectors

A gene fragment corresponding to amino acid residues 65–286 of HA1 from A/California/07/2009 (H1N1) (GenBank accession ACP44189.1) with seven designed mutations was synthesized (GenScript, NJ, USA) and cloned into the pPNLS yeast display vector between the *Sfi*I restriction-sites in-frame with the endogenous yeast signal peptide and aga2 gene at the N-terminal end and c-Myc tag at the C-terminal end [15]. This construct will be referred to as pPNLS-H1pHA9. In the pPNLS-H1pHA9 background, four mutations were introduced to disrupt the Sa-antigenic site of A/California/07/2009 (H1N1) HA. Alternatively, the Sb-antigenic site of A/California/07/2009 (H1N1) HA was disrupted by introducing six mutations 6,10. These constructs have been referred to as pPNLS-H1pHA9(Sa) and pPNLS-H1pHA9(Sb), respectively. The sequences of all the constructs are given in the Supplementary information.

#### iii) Yeast surface display of HA1 fragments and antibody labeling experiments

The pPNLS yeast display plasmids [pPNLS-H1pHA9, H1pHA9(Sa) and H1pHA9(Sb)] were transformed into the yeast strain EBY100 using lithium acetate (LiAc)/single-stranded DNA (SS-DNA) (LiAc/SS)-carrier DNA/PEG method as described previously [16]. The display plasmid contains a tryptophan marker gene (TRP1) that allows screening of the transformed yeast on a selection media lacking tryptophan. Expression of the HA1 proteins on the yeast cell surface was performed as previously described [17]. Briefly, a single, well-isolated, transformed colony was inoculated into 3 ml of synthetic dextrose casamino acids (SDCAA) media (20 g/L dextrose, 6.7 g/L Difco yeast nitrogen base, 5 g/L Bacto casamino acids, 14.7 g/L sodium citrate, 4.29 g/L Citric acid; pH 4.5) and grown at 30°C under shaking conditions until an OD_600_ of 2–3 was reached. Yeast cells were then induced for protein display by transferring to synthetic galactose casamino acids (SGCAA) medium (same as SDCAA medium except 20 g/L galactose instead of dextrose) and incubated with shaking at 20°C for 24 h.

After induction, a total of 1×10^6^ yeast cells were washed with PBS containing 1% BSA (PBSB). Surface expression of the displayed HA1 fragments was probed using anti-c-Myc chicken antibody (Life technologies, NY, USA) at a pre-determined dilution (1∶300 in PBSB; 1 h, 4°C) directed towards the C-terminal tag and subsequently stained with Alexa Fluor-488 goat anti-chicken antibody (Life technologies) at a pre-determined dilution (1∶300 in PBSB; 1 h, 4°C, protected from light). The conformational integrity of displayed protein was detected by binding to MAb 2D1. 2D1-labeled yeast cells were subsequently detected by staining with goat anti-human phycoerythrin (PE) (Sigma Aldrich Inc.) at a pre-determined dilution (1∶100 in PBSB; 1 h, 4°C). For binding specificity of surface-expressed HA1 fragments, the induced cells were incubated with different concentrations of MA2077 (1 h, 4°C), and subsequently stained with Alexa Fluor-633 rabbit anti-mouse antibody (Life technologies, NY, USA) at a pre-determined dilution (1∶1500 in PBSB; 1 h, 4°C). After washing the stained cells with PBSB, fluorescence was detected with a flow cytometer (BD FACS-Canto, BD Biosciences, NJ, USA) and further analyzed with BD FACSDiva cytometry software (BD Biosciences).

## Results

### Generation and characterization of MAbs against pandemic H1N1

**Figure 1:**
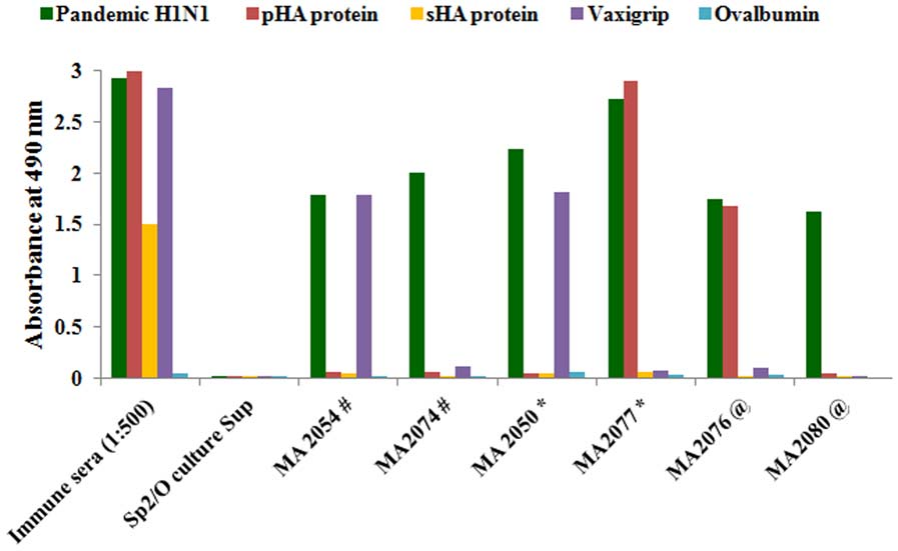
ELISA reactivity profile of MAbs generated against pandemic H1N1. Culture supernatants of various hybrid cell clones generated against pandemic H1N1 virus were tested in ELISA against pandemic H1N1 inactivated virus, Vaxigrip (2009–10) and ovalbumin as negative control (200 ng/well). Additionally, plates coated with pandemic H1N1 A/California/04/2009 rHA protein (pHA) and seasonal H1N1 A/Solomon Islands/03/2006) rHA protein (sHA) (160 ng/well) were also employed. Sera at a dilution of 1∶500 from one of the mice immunized with the inactivated pandemic H1N1 virus was used as positive control and Sp2/O culture supernatant was used as a negative control. Representative reactivity profiles of six MAbs are shown. MAbs marked “#” are of IgM isotype, “*” IgG1 isotype and “@” IgG2a isotype, respectively.

**Figure 2:**
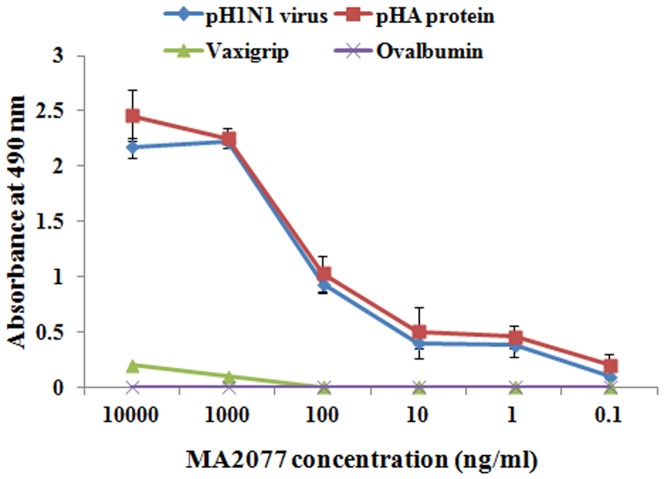
ELISA reactivity profile of MA2077. Purified MA2077 was tested at varying concentrations in ELISA with pandemic H1N1 inactivated virus, Vaxigrip (2009–10 season, Sanofi Pasteur), ovalbumin as negative control (200 ng/well) and mammalian expressed recombinant pandemic H1N1 A/California/04/2009 HA protein (pHA; 160 ng/well).

Employing the protocol as described in ‘*Materials and Methods*’, 14 MAbs were generated against pandemic H1N1 virus. Five MAbs were of IgM isotype, 7 were of IgG1 isotype and 2 MAbs were of IgG2a isotype. The ELISA reactivity profile of 6 representative MAbs is shown [Fig. 1]. In ELISA, 2 MAbs, designated as MA2074 and MA2080 reacted with pandemic H1N1 virus whereas MA2076 and MA2077, in addition also reacted with rHA of pandemic H1N1 (A/California/04/2009) [Fig. 1]. All these 4 MAbs failed to react with Vaxigrip (2009–10) suggesting their specificity for pandemic H1N1 virus. Ten MAbs also showed reactivity with Vaxigrip (2009–10) whereas none of the MAbs reacted with rHA of seasonal H1N1 (A/Solomon Islands/03/2006) and ovalbumin, used as negative control. The culture supernatants of 2 MAbs (MA2076 and MA2077) reacting with the pandemic H1N1 rHA were tested in a microneutralization assay with homologous virus. One of the IgG1 isotype MAb, MA2077, showed neutralization (∼100%) of 100 TCID_50_ dose of the pandemic H1N1 virus (data not shown). To obtain higher amounts of the antibody, hybrid cell clone secreting MA2077 was further grown as ascites and antibody was purified using Protein-G. Purified MA2077 was again tested in ELISA and showed significant binding upto 1 ng/ml with pandemic H1N1 virus as well as pandemic H1N1 rHA (A/California/04/2009) protein and failed to react with Vaxigrip (2009–10, Sanofi Pasteur) and ovalbumin (Fig. 2).

### Binding studies on Biacore

**Figure 3:**
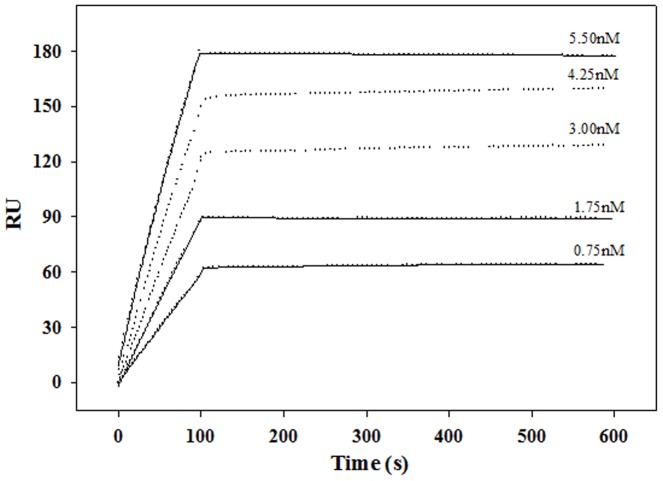
Biacore binding studies. 800 resonance units (RU) of purified MAb MA2077 was immobilized on the surface of a CM5-chip. As indicated, different concentrations of the pandemic H1N1 A/California/04/2009 rHA trimer was passed over each channel. A channel immobilized with ovalbumin served as negative control. MA2077 binds to pandemic rHA with picomolar afiinity (*K*_D_ = 2.1±0.4 pM). The kinetic parameters were obtained by fitting the data to 1∶1 Langmuir interaction model using BIA EVALUATION 3.1 software. The dissociation phase for two concentrations (4.25 nM and 3.0 nM) could not be fitted since there was no measurable change in response units (RU). The data points are in solid circles, while the fits are shown by solid lines. No binding between rHA (H1N1) A/Puerto Rico/8/34 and MA2077 could be detected (sensograms not shown).

MAb MA2077 bound rHA (pandemic H1N1) A/California/04/2009 with very high affinity and specificity. The equilibrium dissociation constant (*K*_D_) of binding of MA2077 to pandemic HA was 2.1±0.4 pM (Fig. 3, Table 1). There is minimal change in response units (RU) during the dissociation phase probably because of a very tight complex between the antibody-antigen (MA2077-pandemic HA), hence, at best, we can determine apparent off-rates [*k*_off_ (1/s) = 9.27×10^−6^]. MA2077 is specific to the pandemic HA as the antibody did not show any detectable binding to rHA (H1N1) A/Puerto Rico/8/34 (Table 1).

**Table 1:** Kinetic parameters for binding of MAb MA2077 to rHA by surface plasmon resonance.

Analyte	*k*_on_ (1/Ms)	*k*_off_ (1/s)	*K*_D_ (M)
A/California/04/2009 HA (H1N1)	4.25×10^6^	9.27×10^−6^	2.1×10^−12^ ±0.4
A/Puerto Rico/8/34 HA (H1N1)	-a	-a	-a

MA2077 was immobilized on the surface of a CM5-chip and analytes were passed over this surface at different concentrations.

Reported values are the mean of kinetic parameters obtained at different concentrations.

a No detectable binding.

### MAb MA2077 potently neutralizes pandemic H1N1 virus

Purified MA2077 was tested in an *in vitro* microneutralization assay against 2009 pandemic H1N1 virus as well as seasonal H1N1 (A/Solomon Islands/03/2006) virus. The IC_50_ and specific activity values were calculated as described in *Materials and Methods*. MA2077 showed potent neutralization of pandemic H1N1 virus with an IC_50_ of 0.08 µg/ml. The specific activity of MA2077 against pandemic H1N1 virus was as low as 0.045 µg/ml (Table 2). However, MA2077 failed to neutralize seasonal H1N1 virus (A/Solomon Islands/03/2006) upto 25 µg/ml, the highest concentration tested. To examine whether MA2077 inhibits the influenza virus by blocking its attachment to the sialic acid receptor on the host cell-surface, HI assay was performed using 1% guinea pig RBCs and 4 HA units of the virus. MA2077 showed HI activity against the pandemic H1N1 virus with specific activity of 0.50 µg/ml, whereas it failed to show HI activity against seasonal H1N1 virus (A/Solomon Islands/03/2006) at concentrations upto 25 µg/ml (Table 2).

**Table 2:** *In vitro* neutralization and HI activity of MA2077.

Virus strain	Test	IC_50_ value (µg/ml)	Specific activity (µg/ml)^a^
Pandemic H1N1 (A/California/07/2009)	Neutralization assay	0.08	0.045
	HI assay	NA	0.50
Seasonal H1N1 (A/Solomon Islands/03/2006)	Neutralization assay	>25	>25
	HI assay	NA	>25

a Specific activity of MAb is the minimum concentration of the antibody required to neutralize 10% of 100 TCID_50_ dose of the virus in an *in vitro* microneutralization assay or to inhibit 4 HA units of the virus in HI assay using 1% guinea pig RBCs.

>25 indicates that inhibitory activity was not detected at any concentration upto 25 µg/ml.

NA- Not applicable.

### MAb MA2077 competes with MAb 2D1 for binding on pandemic H1N1 HA

**Figure 4:**
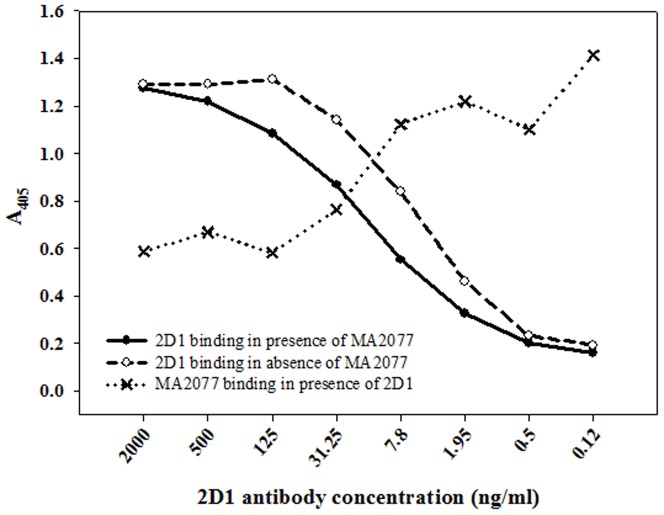
Competition ELISA between MAb MA2077 and 2D1. Microtiter plates were coated with pandemic H1N1 A/California/04/2009 rHA (125 ng/well) and subsequently incubated with varying concentrations of MAb 2D1. Competition ELISA was performed by adding a fixed concentration of MA2077 (1 ng/ml) and plates were processed as described in *Materials and Methods*. Values are expressed as mean of the absorbance of duplicate readings obtained at 405 nm.

2D1, a previously reported MAb, isolated from a survivor of the 1918 Spanish flu binds within the ‘Sa’ antigenic site of 2009 pandemic H1N1 HA [10]. Competition ELISA between MA2077 and 2D1 was done to determine, if the two antibodies bind to sterically overlapping epitopes on pandemic H1N1 HA. The concentration of MAb MA2077 used for competition was determined from the titration curve with pandemic H1N1 rHA (Fig. S1). In the presence of MA2077, binding of 2D1 to rHA of pandemic H1N1 (A/California/04/2009) was significantly decreased when compared with the binding of 2D1 to rHA in the absence of any competitor (MA2077). Concomitantly, with decreasing 2D1 concentrations the binding of MA2077 to pandemic H1N1 rHA increased (Fig. 4). This data suggests that residues within the ‘Sa’ antigenic site may contribute to the epitope of MA2077.

### Design of head domain fragments of 2009 pandemic H1N1 HA

**Figure 5:**
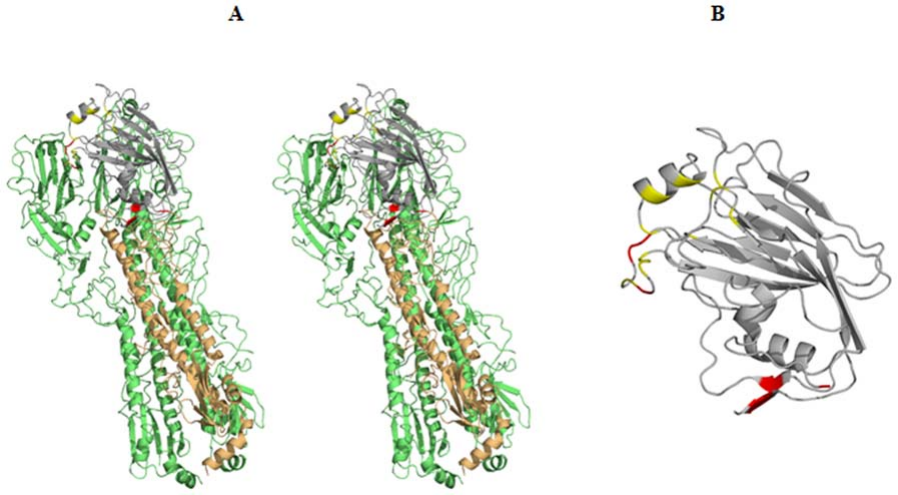
Design of H1pHA9 fragment. An HA1 fragment, H1pHA9, defined by stable breakpoints was designed *in silico*. (A) Shown in stereoview is the cartoon representation of H1N1 A/California/04/2009 HA trimer (PDB ID: 3LZG). Residues 65–286 comprising H1pHA9 (colored grey) have been mapped onto a monomer (orange) of the crystal structure of H1N1 A/California/04/2009 HA. The rest of the molecule is colored green. The mutations introduced in H1pHA9 are colored red. The receptor binding site residues are colored yellow. (B) The mutated residues (red) in H1pHA9 do not contribute to any of the antigenic sites or the receptor binding site (yellow) on HA. The figures were generated in PyMOL (The PyMOL Molecular Graphics System, version 1.2r2, DeLano Scientific, LLC).

An HA1 fragment, H1pHA9, defined by stable breakpoints in the protein structure was designed *in silico* (Fig. 5A). Mutations were introduced in the designed fragment to remove exposed hydrophobic patches generated due to interactions lost with the rest of HA, which could potentially cause protein aggregation. A similar approach has been previously used by us to design stable influenza and HIV-immunogens and inhibitors [18]–[20]. These mutations were chosen based on accessibility calculations on a homology model of A/California/07/2009 (H1N1) HA sequence (GenBank accession ACP44189.1) using the structure of A/South Carolina/1/1918 (H1N1) HA [PDB ID: 1RD8] as template which was available when this study was initiated [21]. These calculations have been subsequently validated using the crystal structure co-ordinates of A/California/04/2009 (H1N1) HA [PDB ID: 3LZG] which became available later [10]. Wild-type residue V97 mutated in our construct, is buried in the native A/California/04/2009 (H1N1) HA structure, however we do not expect an iso-steric substitution to Thr to considerably affect the folding of our designed fragment. The mutated residues do not contribute to any previously characterized antigenic sites or the receptor binding site on HA. The mutations incorporated in H1pHA9 were A65S, V97T, A232T, I233Q, V237T, I283R, I284T (Fig. 5B, Supplementary Fig. S2). During the course of our work, design of similar HA1-fragments has been reported [22], [23]. However, none of the previously reported designs had these additional engineered mutations.

**Figure 6:**
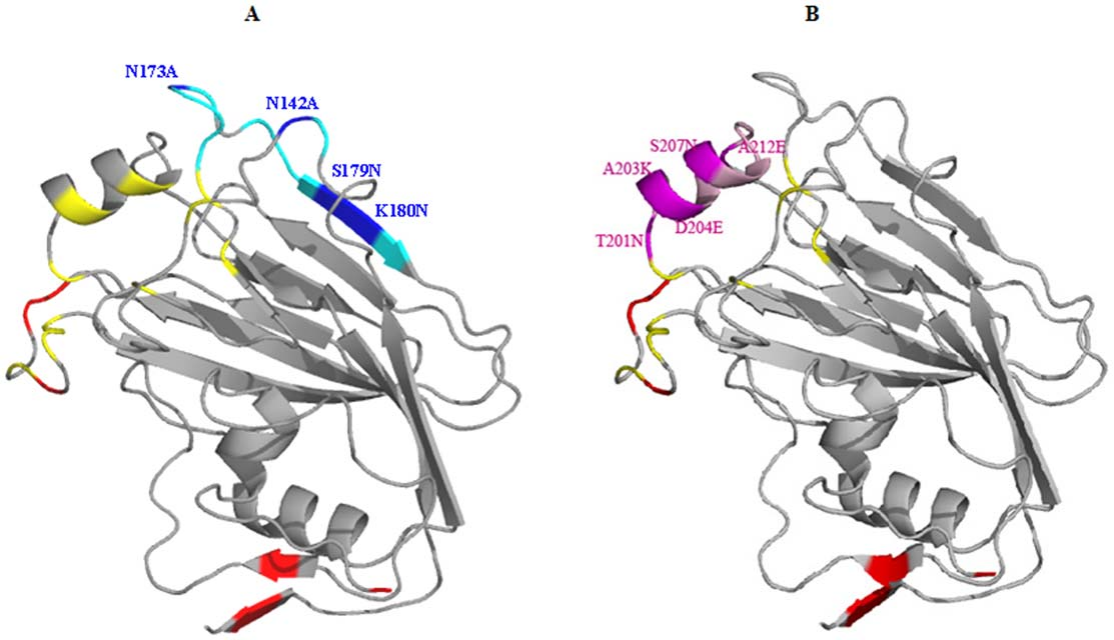
Design of H1pHA9(Sa) and H1pHA9(Sb) constructs. The ‘Sa’ and ‘Sb’ antigenic sites were independently mutated in our designed fragment, H1pHA9. (A) ‘Sa’ antigenic site residues are colored cyan. The mutations introduced in H1pHA9(Sa) to disrupt this epitope are labeled (colored blue). (B) ‘Sb’ antigenic site residues are colored light pink. The mutations introduced in H1pHA9(Sb) are labeled (colored pink). (A, B) Mutations introduced in H1pHA9 to remove exposed hydrophobic patches are in red. The receptor binding site residues are in yellow. The figures were generated in PyMOL (The PyMOL Molecular Graphics System, version 1.2r2, DeLano Scientific, LLC).

The antigenic sites ‘Sa’ or ‘Sb’ of pandemic H1N1 HA in our designed fragment, H1pHA9, were independently mutated to characterize the epitope of MAb MA2077. Residues in these antigenic sites which had an accessibility of >20% were identified and labeled ‘exposed’ [24]. Six residues (one in the Sa-epitope and five in the Sb-epitope) within this ‘exposed’ set which were not conserved between A/California/07/2009 (H1N1) HA (GenBank accession ACP44189.1) and A/Puerto Rico/8/1934 (H1N1) HA (GenBank accession ABD77675.1) were mutated with the wild-type residue from the divergent PR-8 (H1N1) HA sequence. Two other ‘exposed’ residues in the Sa-epitope were mutated to Ala and S179 was mutated to Asn. In total, four mutations were introduced in H1pHA9 to disrupt the Sa-epitope. The mutations incorporated in H1pHA9(Sa) were N142A, N173A, S179N, K180N (Fig. 6A). The Sb-epitope of H1pHA9 was disrupted by introducing six mutations; T201N, A203K, D204E, Q206T, S207N, A212E (Fig. 6B). The sequences of the various constructs are listed in supplementary Fig. S2.

### Epitope mapping by yeast cell-surface display

**Figure 7:**
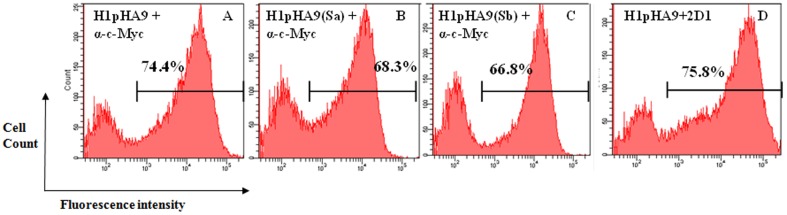
Expression of designed HA1 constructs on yeast cell surface. (A–C) Histograms depicting the fluorescence signal of Alexa Fluor-488 labeled antibody bound to the C-terminal c-Myc tag of the displayed proteins. The Alexa Fluor-488 signal indicates the cell-surface expression of HA1 fragments. (D) The designed fragment, H1pHA9, is well folded and bound the conformationally sensitive MAb 2D1 which was detected with PE-labeled antibody.

**Figure 8:**
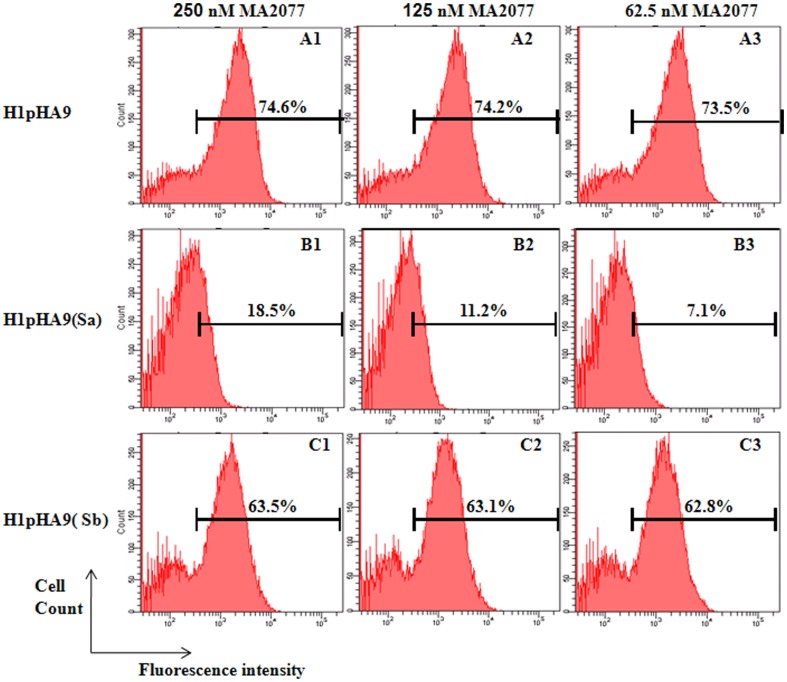
Epitope mapping of MA2077 using yeast cell surface displayed HA1 fragments. Binding of the yeast cell surface displayed H1pHA9 (A1–A3), H1pHA9(Sa) (B1–B3) and H1pHA9(Sb) (C1–C3) proteins to MA2077 was determined at different concentrations of antibody as indicated. The bound MA2077 was stained with Alexa Fluor-633 labeled antibody. The fluorescence histograms suggest that H1pHA9 and H1pHA9(Sb) bind MA2077 with similar affinity. (B1–3) Mutations in the Sa-antigenic site almost completely abolish binding to MA2077.

Here we describe a method to specifically detect if a given MAb is targeted to the ‘Sa’ or ‘Sb’ antigenic sites of the pandemic H1N1 HA. All the HA1-fragments H1pHA9, H1pHA9(Sa) and H1pHA9(Sb) were well expressed on the yeast cell surface, as indicated by the fluorescence histograms for c-Myc labeling (Fig. 7A–C). H1pHA9 displayed on yeast surface was properly folded as assayed with MAb 2D1, which binds to a conformational epitope on HA [10] (Fig. 7D). The yeast cells displaying HA1-fragments were probed with different concentrations of MA2077. The fluorescence histograms for MA2077 labeling suggests that both H1pHA9 and H1pHA9(Sb) bind the neutralizing antibody MA2077, with similar affinity (Fig. 8 A1–3, C1–3). However, H1pHA9(Sa) has a drastically reduced affinity for MA2077, and at the lowest concentration of MA2077 (62.5 nM) tested, there is negligible binding (Fig. 8 B1–3).

## Discussion

Considering the public health significance of the emergence of new pathogens like pandemic H1N1, it is imperative to search for newer diagnostic and therapeutic modalities to contain/alleviate the disease progression. MAbs have been reported recently which can broadly neutralize different influenza virus strains including pandemic H1N1 [25], [26]. In spite of this, there is scope to generate a MAb with higher affinity, specificity and neutralizing ability that could find utility in sensitive diagnostics and therapeutics due to reduced dosage. The animal immune system has been naturally designed for selection of high affinity full length MAbs as compared to *in vitro* antibody generation approaches such as phage display methods which result in scFv that is subsequently converted to full length IgG. Generation of murine MAbs is also cost effective. Hence, in this direction, we have generated murine MAbs against the pandemic H1N1 virus. Two of the MAbs specific to pandemic H1N1 virus also reacted with pandemic H1N1 (A/California/04/2009) rHA protein. Some of the other MAbs also showed reactivity with Vaxigrip (2009–10 Season, Sanofi Pasteur) and thus it is possible that these antibodies either recognized proteins other than HA, such as neuraminidase or matrix protein of the pandemic H1N1 virus. The potential of such MAbs in developing diagnostic tools for detection of pandemic and seasonal influenza virus infection can be explored. One of the MAb, MA2077 specifically recognized pandemic H1N1 (both whole virus protein and HA protein) with significant reactivity upto 1 ng/ml. MA2077 bound pandemic H1N1 rHA (A/California/04/2009) with very high affinity (*K*_D_ = 2.1±0.4 pM) and specificity as determined by surface plasmon resonance.

Unlike MA2077, culture supernatant of the other MAb (MA2076) reactive with the pandemic H1N1 rHA, failed to neutralize the homologous virus (data not shown). It can be speculated that MA2076 binds to non-neutralizing epitopes of the HA protein. MA2077 neutralized pandemic H1N1 virus in an *in vitro* microneutralization assay with an IC_50_ as low as 0.08 µg/ml. Burioni *et al.*, have reported pandemic H1N1 neutralizing MAbs with IC_50_ in the range of 2.8–4 µg/ml [27]. Cabral *et al.*, have also been able to isolate neutralizing MAbs against pandemic H1N1 virus, with IC_50_ as low as 0.0025 µg/ml, wherein neutralization capacity was assessed by employing plaque reduction assay instead of microneutralization assay [28]. Hence, to the best of our knowledge, MA2077, is a potent neutralizing MAb against pandemic H1N1 virus, reported so far. Further, MA2077 also showed HI activity against the pandemic H1N1 virus (Table 1). This suggested that antibody mediates neutralization by binding to the HA1 head region of the HA protein and blocking its attachment with the sialic acid receptor. HI test is a routinely used assay for identification of isolates of influenza viruses. Therefore antibodies like MA2077 with high specificity may find its application in influenza surveillance.

After establishing the ability of MA2077 to neutralize H1N1 by binding to HA protein, the epitope recognized by it has been elucidated by competition ELISA with a human monoclonal antibody, 2D1. The MAb 2D1 isolated from a survivor of 1918 Spanish flu, binds to both 1918 as well as 2009 pandemic H1N1 viruses by recognizing the conserved ‘Sa’ antigenic site and has reported specific activity of 0.04 µg/ml [9], [10]. Competition results showed that as the concentration of MAb 2D1 decreased, binding of MA2077 to rHA increased (Fig. 4). This suggested that the two antibodies might be binding to overlapping epitopes on pandemic H1N1 HA.

Yeast cell-surface display is a convenient method for expression and screening of combinatorial libraries of engineered proteins. We have developed a methodology to rapidly characterize HA head-domain specific antibodies using yeast cell-surface display of HA1 fragments. A similar approach has been previously adopted to characterize EGFR-specific antibodies [29]. The full length protein (HA0) and its subunits (HA1 and HA2) from a highly pathogenic H5N1 influenza virus have been previously displayed on the yeast surface to map the antibody response against H5N1 HA [30]. In the present work, we designed a construct (H1pHA9) encompassing the HA1 region corresponding to amino acid residues 65–286 of A/California/07/2009 (H1N1) HA (GenBank accession ACP44189.1). This construct (H1pHA9) was expressed on the yeast cell surface. H1pHA9 showed good expression on the yeast cell surface, and bound well to two conformationally sensitive MAbs, 2D1 and MA2077 (Fig. 7D, Fig. 8 A1–3). Though ‘Sa’ and ‘Sb’ sites are spatially in close proximity, simultaneous binding of antibodies to these sites is not possible [6]. Hence, to demarcate the involvement of either the ‘Sa’ or ‘Sb’ site in MA2077 binding, mutations were independently introduced in both ‘Sa’ and ‘Sb’ sites of our construct H1pHA9 (Fig. 6A–B). The expression levels of both H1pHA9(Sa) and H1pHA9(Sb) were comparable to that of H1pHA9. However, MA2077 binding to H1pHA9(Sa) displayed on yeast surface was completely abolished due to the introduced mutations (Fig. 8 B1–3). However, binding to H1pHA9(Sb) remained uncompromised (Fig. 8 C1–3). This data further confirmed that the MA2077 binds within the ‘Sa’ antigenic site. Interestingly, mouse MAbs have also been generated against 1918 Spanish flu virus-based vaccine, which neutralized the pandemic H1N1 virus and its epitope has also been mapped to ‘Sa’ antigenic site [31].

Acquisition of glycosylation at immunodominant sites is one of the strategies adopted by influenza viruses to evade the host immune response. Phylogenetic analyses have shown that the 1918 H1N1 virus (A/South Carolina/1/1918) lacked glycosylation near the ‘Sa’ site and H1N1 influenza viruses from 1930–2007 have gradually incorporated glycosylation at and around this site. Interestingly, the 2009 pandemic H1N1 virus also lacks glycosylation at or near the ‘Sa’ antigenic site [10]. This may have facilitated elicitation of antibodies recognizing the ‘Sa’ upon immunization. Sequence comparison of variable regions of MA2077 and 2D1 showed an identity of less than 25% (data not shown). Further studies regarding the interaction of MA2077 with its epitope may reveal additional underlying mechanisms employed by such antibodies binding to ‘Sa’ site to neutralize the infectivity of the virus.

Due to its high affinity and specificity, MA2077 can be a potential tool for sensitive diagnosis of pandemic H1N1 virus; for studying the mechanistic details of antigen-antibody interactions in context of neutralization of influenza virus and upon humanization may be considered for therapeutic purposes. However, recent reports have shown that under immune selection pressure, escape mutants readily emerge for pandemic H1N1, and some of the mutations lie in the ‘Sa’ region [32], [33]. Hence, to take care of the escape mutants, it will be imperative to use this antibody in combination with other available MAbs for diagnosis of H1N1 influenza virus or as therapeutics. One of the unique merits of the present study is that we have developed a yeast-display based technique for rapidly identifying the putative epitope for head-directed (Sa/Sb) antibodies using flow cytometry. This technique can be further fine-tuned to obtain residue-specific information.

## Supporting Information

Figure S1 **Titration curve of MAbs MA2077 and 2D1 against pandemic H1N1 rHA by direct-binding ELISA.** Varying concentrations of MA2077 and 2D1 were tested against mammalian cell expressed pandemic H1N1 A/California/04/2007 rHA (125 ng/well) in direct binding ELISA. Values are expressed as the mean of triplicate readings of absorbance at 405 nm.
(TIF)

Figure S2 **Sequence of the designed HA1-fragments.** H1pHA9 corresponds to residues 65–286 of HA1 from A/California/07/2009 (H1N1) (GenBank accession ACP44189.1) with seven designed mutations to remove exposed hydrophobic patches. The mutated residues are highlighted by bold alphabets. Four additional mutations were made in H1pHA9(Sa) to disrupt the ‘Sa’ antigenic site. These residues are underlined. The ‘Sb’ antigenic site was disrupted in H1pHA9(Sb) by six mutations in H1pHA9. These residues are italicized. All the HA1-fragments were cloned into the pPNLS yeast display vector between the SfiI restriction-sites in-frame with the endogenous yeast signal peptide and aga2 gene at the N-terminal end and c-Myc tag at the C-terminal end.
(TIF)
