# Supplementary figures and images for: Isolation of a High Affinity Neutralizing Monoclonal Antibody against 2009 Pandemic H1N1 Virus That Binds at the ‘Sa’ Antigenic Site
Source: PLoS One. 2013 Jan 31;8(1):e55516. doi: 10.1371/journal.pone.0055516 (PMC3561186; doi:10.1371/journal.pone.0055516)

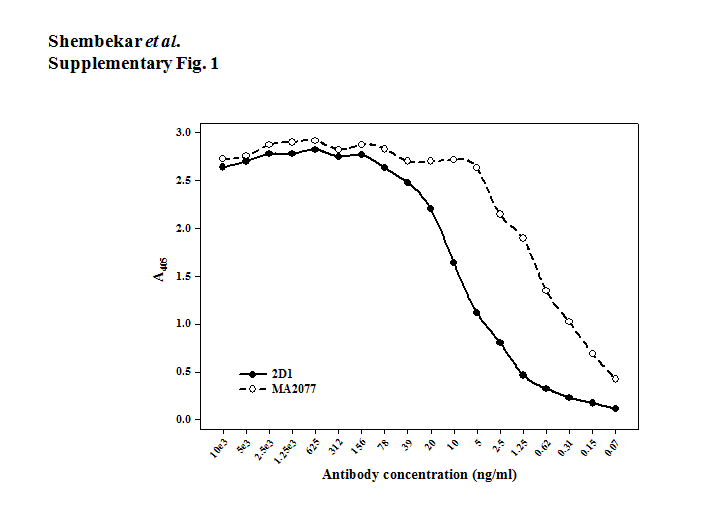

Supplement: Figure S1 — Titration curve of MAbs MA2077 and 2D1 against pandemic H1N1 rHA by direct-binding ELISA. Varying concentrations of MA2077 and 2D1 were tested against mammalian cell expressed pandemic H1N1 A/California/04/2007 rHA (125 ng/well) in direct binding ELISA. Values are expressed as the mean of triplicate readings of absorbance at 405 nm. (TIF) [file pone.0055516.s001.tif]

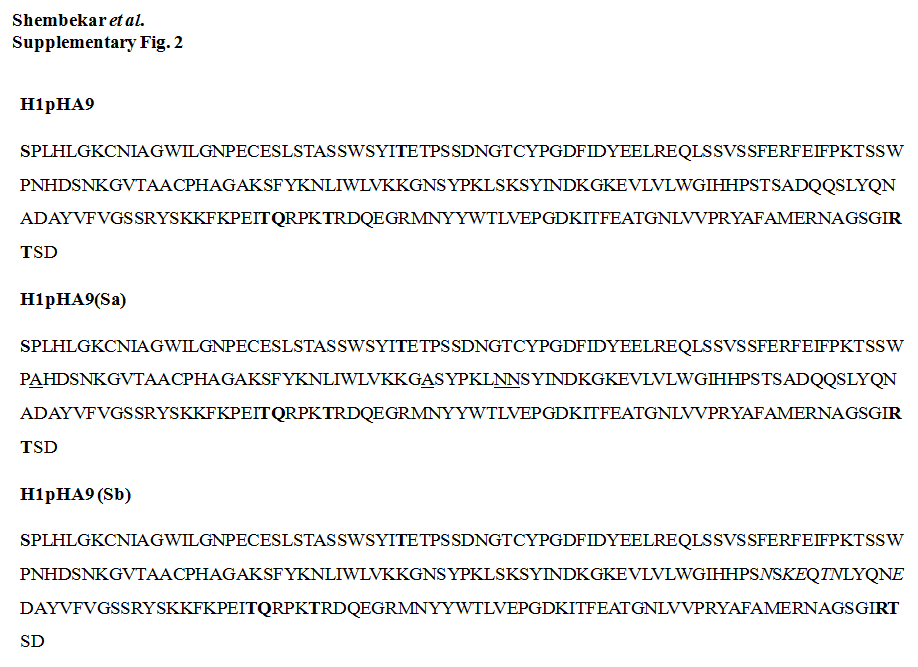

Supplement: Figure S2 — Sequence of the designed HA1-fragments. H1pHA9 corresponds to residues 65–286 of HA1 from A/California/07/2009 (H1N1) (GenBank accession ACP44189.1) with seven designed mutations to remove exposed hydrophobic patches. The mutated residues are highlighted by bold alphabets. Four additional mutations were made in H1pHA9(Sa) to disrupt the ‘Sa’ antigenic site. These residues are underlined. The ‘Sb’ antigenic site was disrupted in H1pHA9(Sb) by six mutations in H1pHA9. These residues are italicized. All the HA1-fragments were cloned into the pPNLS yeast display vector between the SfiI restriction-sites in-frame with the endogenous yeast signal peptide and aga2 gene at the N-terminal end and c-Myc tag at the C-terminal end. (TIF) [file pone.0055516.s002.tif]
